# Symptomatic hypercalcemia in a patient with chronic tophaceous gout: a case report

**DOI:** 10.1186/1757-1626-1-72

**Published:** 2008-08-07

**Authors:** Alok Sachdeva, Bruce E Goeckeritz, Alyce M Oliver

**Affiliations:** 1 Dept. of Rheumatology, Medical College of Georgia, Augusta, Georgia, USA

## Abstract

Hypercalcemia has been widely associated with granulomatous processes. This is due to enhanced extra-renal conversion of calcidiol to calcitriol by activated macrophages within the granuloma. Symptomatic hypercalcemia due to granulomatous disorders is not common, with the incidence in sarcoidosis ranging from 10–20%. Large aggregates of monosodium urate crystals in patients with longstanding chronic tophaceous gout can serve as the inciting antigen for the development of granuloma, but hypercalcemia has not been described in this context. We report a case of symptomatic hypercalcemia due to gouty tophi induced granulomatous inflammation. Long term treatment with immunosuppressants, in addition to bisphosphonates and uric acid lowering therapy, has led to stabilization of serum calcium levels and other lab parameters indicative of granulomatous burden.

## Case presentation

**Figure 1:**
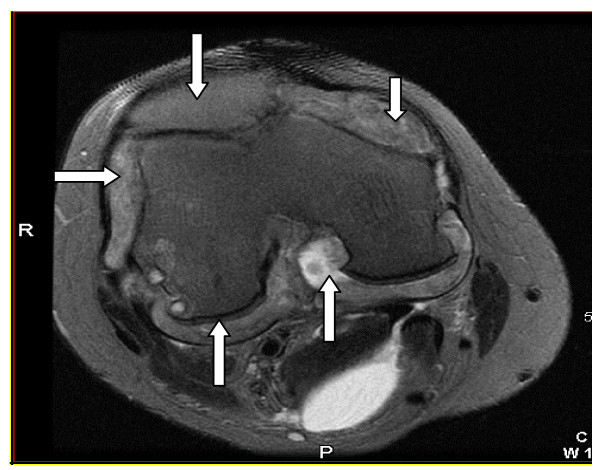
Fat suppressed T2 weighted axial image of the right knee showing an inflammatory pannus within the syovial capsule.

**Figure 2:**
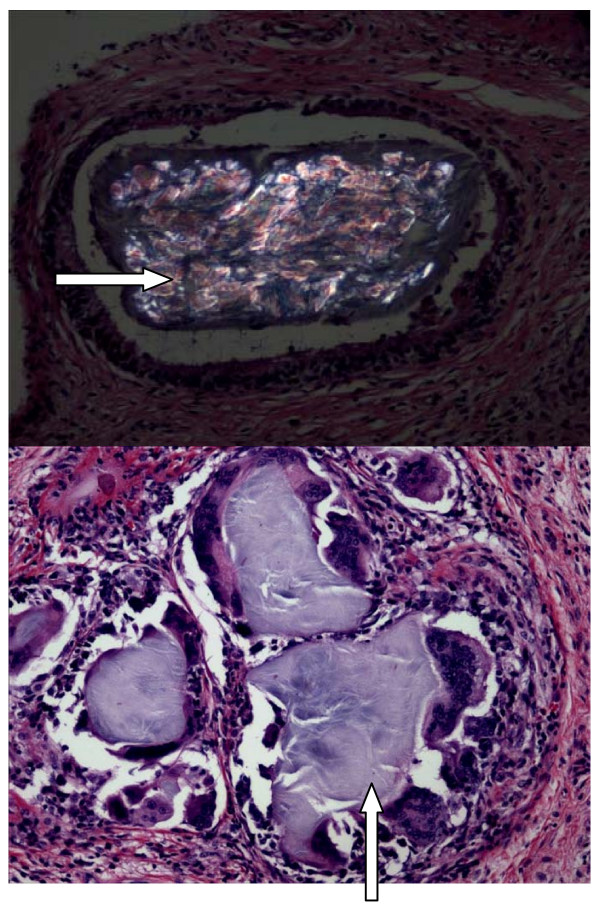
Syonvial biopsy of right knee showing proliferative chronic synovitis with granulomatous inflammation and giant cells containing tophaceous material (arrow). Polarized microscopy was consistent with gout.

A 41-year-old Caucasian male whose past medical history was significant only for chronic tophaceous gout, for which he was on allopurinol and colchicine, was seen for worsening right knee pain with mechanical symptoms of locking and buckling. An MRI of the right knee was obtained to evaluate for evidence of internal derangement. This revealed an inflammatory mass seen within the synovial capsule causing extensive erosions of the femoral condyles and tibial plateaus (figure 1). The differential diagnosis included pigmented villonodular synovitis versus gouty tophus. Arthroscopically guided synovectomy and biopsy of the mass demonstrated a proliferative chronic synovitis with granulomatous inflammation and giant cells containing tophaceous material. Polarized microscopy revealed strongly negative birefringent needle shaped crystals within the granuloma consistent with gout (figure 2). Pathology was negative for malignancy, did not reveal hemosiderin deposition, and staining and cultures of the specimen did not reveal an infectious cause for the granulomatous inflammation.

**Figure 3:**
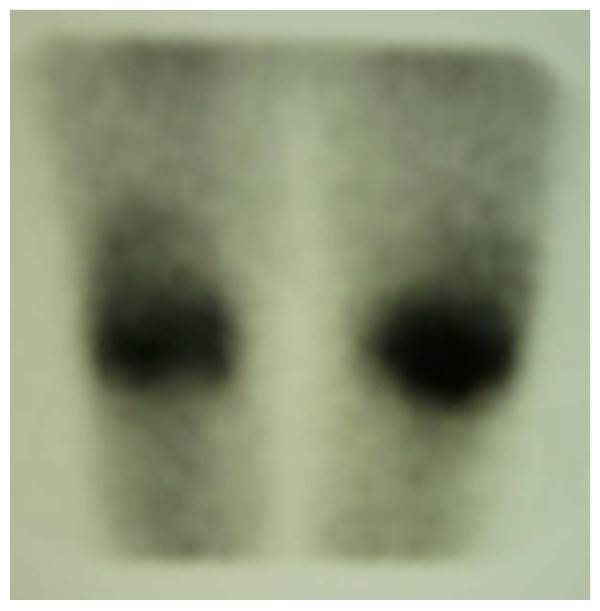
Gallium-67 scan showing increased radiotracer uptake within bilateral knee.

A few months later he presented to the emergency department with complaints of weakness, dizziness, constipation and loss of appetite with weight loss. He was noted to have a serum calcium of 13.5 mg/dl (normal 8.2–10.7 mg/dl) and a ionized calcium of 7.1 mg/dl (normal 4.5–5.3 mg/dl). He was admitted to the hospital for symptomatic hypercalcemia and work up was significant for normal values of prostate specific antigen, cortisol, thyroid stimulating hormone, 25 hydroxyviamin D and parathyroid hormone-related peptide (PTHrp). Both serum and urine protein electrophoresis were normal. Tuberculin skin testing was non-reactive. Chest x-ray did not show hilar adenopathy or interstitial or alveolar infiltrates. MRI of the abdomen and pelvis and three phase bone scan were negative for malignancy or metastatic disease. Blood, urine, and sputum cultures were all negative for bacterial or fungal infections and, as previously mentioned, staining and culture of the previous synovial biopsy specimen did not reveal bacterial or fungal elements. Intact parathyroid hormone (iPTH), however, was noted to be markedly decreased at < 3 pg/ml and both 1,25 dihydroyxvitamin D and angiotensin converting enzyme (ACE) levels were markedly elevated at 110 pg/ml (normal 22–67 pg/ml) and 153 units/l (normal 7–46 units/l) respectively. Gallium-67 scan exhibited intense radiotracer uptake within the knees, left shoulder, and feet (figure 3), which were all areas of previous gouty arthritis involvement. He was therefore thought to have hypercalcemia secondary to granulomatous inflammation induced by gouty tophi. Treatment during hospitalization with intravenous fluids, bisphosphonates, calcitonin, and high dose steroids dramatically decreased serum and ionized calcium levels to within the normal range. The patient's presenting symptoms also resolved and he was discharged home in stable condition with close follow up in our clinic.

## Discussion

If patients with chronic hyperuricemia are left untreated, gouty tophi can develop and usually deposit in subcutaneous tissues. Tophi have also been shown to occur within joints, as was the case in our patient, but have also been shown to deposit in various tissues such as kidney, breast and spinal cord [1]. The histological hallmark of a gouty tophi is created when MSU crystals aggregate with intercrystalline matrix and induce surrounding granuloma formation [2]. The MSU crystals serve as the inciting antigen which leads to an intense inflammatory reaction of macrophages, lymphocytes and large foreign body giant cells which may have completely or partially engulfed the mass of crystals [3].

Hypercalcemia has been described in patients with many types of granulomatous disorders such as tuberculosis, Wegener's granulomatosis, and Crohn's disease. By far the most widely evaluated and one of the more common causes of hypercalcemia due to granulomatous inflammation is sarcoidosis [4,5]. The mechanism of hypercalcemia is PTH-independent extrarenal production of calcitriol from calcidiol by activated mononuclear cells within the granuloma. This leads to increased intestinal absorption of calcium, and to a lesser degree calcitriol-mediated increase in bone resorption [6]. Lab parameters in our patient including elevated 1,25 dihydroxyvitamin D, ACE level, and suppressed iPTH support his granulomatous load as the cause of hypercalcemia [7]. The synovial biopsy suggests that his gouty tophaceous burden is the underlying etiology of the granuloma formation. Furthermore long term use of immunosuppressants, in addition to aggressive uric acid lowering therapy with allopurinol, has maintained serum calcium, 1,25 dihydroxyvitamin D, and ACE levels within normal limits.

This case is unique in that although gouty tophi have been shown to prompt granuloma formation around the aggregation of MSU crystals, to our knowledge there is no literature describing the development of symptomatic hypercalcemia as is seen with a variety of other causes of granulomatous disorders. Obtaining a biopsy from another site which revealed increased radiotracer uptake per gallium-67 scan was contemplated. Showing gouty tophi with surrounding granuloma formation in these areas, as was seen in the synovial biopsy of the knee, would be even more suggestive of our theory but would also be highly unethical as he did very well with the above mentioned medical interventions.

## Conclusion

Since gout is a common medical problem invariably seen by all rheumatologists as well as primary care physicians, this association should be taken into account especially if symptomatic hypercalcemia develops in the context of tophaceous gout. The list of antigens initiating granulomatous inflammation and hypercalcemia is a lengthy one, and based on our findings we feel MSU crystals should be added to this list.

## Competing interests

The authors declare that they have no competing interests.

## Authors' contributions

AS and BG both analyzed and interpreted the patient's data. AS was the major contributor in the writing of the manuscript. BG and AO made substantial contribution to the conception and design of the manuscript. Both BG and AO were involved in revising the manuscript for critically important content. All authors have read and approve the final manuscript.

## Consent

Written informed consent was obtained from the patient for publication of this case report and accompanying images. A copy of the written consent is available for review by the Editor-in-Chief of this journal.

## References

[B1] Sharifabad MA, Tzeng J, Gharibshahi S (2006). Mammary Gouty Tophus: A Case Report and Review of the Literature. The Breast Journal.

[B2] Schweyer S, Hemmerlein B, Radzun HJ, Fayyazi A (2000). Contnuous Recruitment, co-expression of tumor necrosis factor alpha and matrix metalloproteinases, and apoptosis of macrophages in gout tophi. Virchows Arch.

[B3] Gout. http://www.emedicine.com/med/topic924.

[B4] Kang ES, Do MY, Park SY, Hur KY, Ahn CW, Cha BS, Lim SK, Kim KR, Lee HC (2005). Hypercalcemia in Hepatic Tuberculosis: A case Report in Korea. Am J Trop Med Hyg.

[B5] Jacobs TP, Bilezikian JP (2005). Clinical Review: Rare Causes of Hypercalcemia. The Journal of Clinical Endocrinology and Metabolism.

[B6] Clinical Manifestations and Diagnosis of Sarcoidosis. http://www.utdol.com.

[B7] Motoyama K, Inaba M, Emoto M, Morii H, Nishizawa Y (2002). Sarcoidosis Initially Manifesting as Symptomatic Hypercalcemia with the Absence of Organic Involvement. Internal Medicine.

